# A Dynamical Systems Explanation of the Hurst Effect and Atmospheric Low-Frequency Variability

**DOI:** 10.1038/srep09068

**Published:** 2015-03-13

**Authors:** Christian L. E. Franzke, Scott M. Osprey, Paolo Davini, Nicholas W. Watkins

**Affiliations:** 1 Meteorological Institute and Center for Earth System Research and Sustainability (CEN), University of Hamburg, Hamburg, Germany; 2 National Centre for Atmospheric Science and Department of Physics, University of Oxford, Oxford, UK; 3 Institute of Atmospheric Sciences and Climate (ISAC-CNR), Torino, Italy; 4 Max-Planck Institute for the Physics of Complex Systems, Dresden, Germany; 5 Centre for the Analysis of Time Series, LSE, London, UK; 6 MCT, Open University, Milton Keynes, UK; 7 CFSA, University of Warwick, Coventry, UK

## Abstract

The Hurst effect plays an important role in many areas such as physics, climate and finance. It describes the anomalous growth of range and constrains the behavior and predictability of these systems. The Hurst effect is frequently taken to be synonymous with Long-Range Dependence (LRD) and is typically assumed to be produced by a stationary stochastic process which has infinite memory. However, infinite memory appears to be at odds with the Markovian nature of most physical laws while the stationarity assumption lacks robustness. Here we use Lorenz's paradigmatic chaotic model to show that regime behavior can also cause the Hurst effect. By giving an alternative, parsimonious, explanation using nonstationary Markovian dynamics, our results question the common belief that the Hurst effect necessarily implies a stationary infinite memory process. We also demonstrate that our results can explain atmospheric variability without the infinite memory previously thought necessary and are consistent with climate model simulations.

Hurst's environmetric observations in the 1950s first sparked interest in the natural phenomenon of anomalously fast growth of rescaled range in hydrological time series, most famously from the Nile river^1^,^2^. Rescaled range is a measure of the variability of a time series and is calculated by dividing the range of the values by the standard deviation. This is done for increasing window sizes which are than averaged^2^,^3^.

These observations of the growth of range of what is now known as the ‘Hurst' effect stimulated much debate, because Feller showed rigorously that for a very general class of finite variance stochastic processes, the rescaled range grows asymptotically with the record length *L* as *L*^1/2^ 4. Many explanations centered on pre-asymptotic effects, but a more mathematically elegant explanation came with the introduction by Mandelbrot, Van Ness and Wallis of fractional Gaussian noise (fGn), the first stationary model which was able to reproduce them. fGn was in itself controversial, however^5^,^6^, because it gained the desirable and tractable property of stationarity at the price of introducing infinite-ranged temporal memory or LRD. LRD implies that in order to predict the next state of a system its whole past is needed. This is different from typical dynamical systems whose next state is determined just by the current state. Such systems are called Markovian. This property appeared to many to be inconsistent with the Markovian nature of the equations of motion.

However Mandelbrot, as early as 1965^5^,^7^ and contemporary with his work on fGn and fractional Brownian motion (fBm), showed that at least one other type of non-stationary model could exhibit the Hurst effect. This other model has not received the same attention as stationary LRD models and raises an important question of what should be used for the modeling of natural systems. To the relatively familiar random walk models such as his own fBm, which is integrated fGn, Mandelbrot added a class of switching models with long tailed distributions for the intervals between the state changes, which he called “conditionally stationary”. Stationary fGn and these two classes of non-stationary models all shared a form of 1/f power spectrum at low frequencies, the signature of self-affinity. The presence of fluctuations on all timescales in 1/f noises complicates inference of trends in short time series, as seen for instance in climate^8^,^9^,^10^ and hydrology^11^. Whether a complex system such as the climate is stationary or non-stationary, and if the latter, what type, thus has significant implications for our ability to perform skillful predictions. However, the very ubiquity of 1/f noise poses a problem, in Mandelbrot's own words that *“reducing the notion of 1/f noise” to “self-affinity … shows it to be very severely under-specified”*^6^, so that other considerations need to be taken into account when choosing a model.

Much empirical model choice has been based on the idea of parsimony (Occam's razor), a principle of model selection which states that one should select the model with the fewest necessary assumptions. Parsimony favors “simplicity” or “elegance”, but these concepts admit different interpretations in different sciences. A more complicated model can turn out to be more skillful^12^, but this skill may be at the expense of insight. In the end, however, whether a complex system is better described by a stationary or non-stationary model may not be decided purely by parsimony. In geosciences physical reservations about the LRD concept, and an apparent lack of awareness that Mandelbrot had already proposed a conditionally stationary alternative^7^, motivated Klemes^13^ to discuss switching models, while Bhattacharya et al.^14^ showed that the presence of trends could produce a Hurst effect under certain conditions. However in econometrics, it has long been recognized by some authors that in addition to the classes of models discussed above, it is essential to consider alternatives which are motivated by parsimonious assumptions about the time series. In particular, Diebold and Inoue^15^ have shown that some Markovian regime switching models can indeed produce 1/f signatures over a wide frequency range, despite not possessing long tailed distributions of times between switching, and have argued that they may be more relevant in some systems. Mesa et al.^16^ have argued on physical grounds for the importance of deterministic low dimensional chaos as an origin for the Hurst effect, in particular focusing on the ‘critically slowed' motion in systems close to a bifurcation.

It is well known that LRD requires slow, algebraic decay of the empirical autocorrelation function; *ρ*(*τ*) ~ *τ*^2*d*−1^ 17,18, but, importantly, it also assumes stationarity^17^, without which a memory extending to *t* = −∞ cannot be defined. When it is observed, the Hurst phenomenon is usually attributed to phenomena which are best described by stochastic processes, such as: self-similar scaling processes, aggregation of short-range dependent stochastic processes, turbulence or the distributional properties of waiting times (see Refs. 7, 17 for more details). The memory parameter *d* is conventionally defined^17^ via the slope of the power spectrum, or alternatively by the slope of the decay of the autocorrelation function, on a doubly logarithmic plot. For d > 0 we talk about persistent time series, where large values tend to be followed by large values and vice versa. For d < 0 we have anti-persistence; i.e. positive values tend to be followed by negative values and vice versa. For d = 0 we have white noise; i.e. no autocorrelation. The widely used autoregressive models approach a white noise power spectrum for large time scales^8^.

Here we show that one of the most seminal models of deterministic chaotic dynamics, Lorenz's 1963 model^19^ which he abstracted from Rayleigh-Benard convection, exhibits the Hurst effect. We show that the origin of the Hurst effect in the Lorenz model is regime behavior. This has implications for our understanding of how the Hurst effect can arise, and for how we interpret observational data in many fields. That the Lorenz 63 model exhibits the Hurst effect is a surprising and counter-intuitive result because deterministic chaotic systems are frequently thought to be white noise on time scales larger than the Lyapunov time scale (e.g. Ref. 20), and they are sometimes used for the generation of independent and identically distributed (iid) random numbers^21^.

## The Paradigmatic Model: Lorenz 63

**Figure 1:**
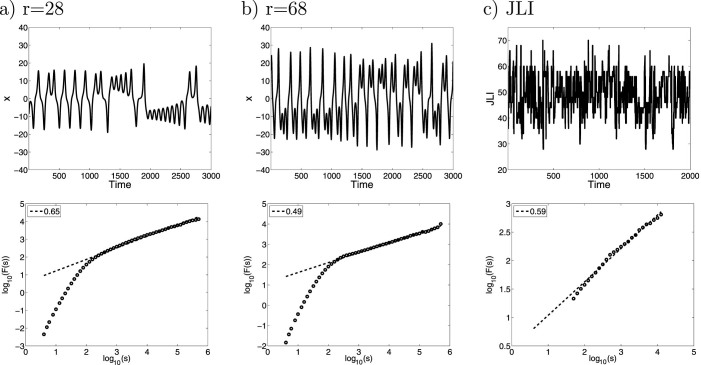
Time series (upper row) and DFA2 (lower row) for a) the Lorenz 63 model with r = 28, b) the Lorenz 63 model with r = 68 and c) the JLI derived from ERA40 reanalysis data^36^. The regression line is trending upward towards lower frequencies for the r = 28 case and the JLI with a slope larger than 0.5 whereas the slope is about 0.5 for the r = 68 case. These results are consistent with the r = 28 case and the JLI exhibiting the Hurst effect and the r = 68 case being white noise. The time series is sampled in 0.1 time units.

The nonlinear deterministic Lorenz 63 model^19^ is given by:
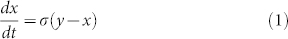

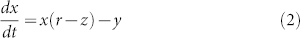

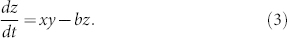
It is the archetypal model for deterministic chaos^19^. We use the standard parameter values, *b* = 8/3 and *σ* = 10 and vary *r*. Fig. 1a shows the typical behavior of the standard Lorenz model with *r* = 28. With these settings the system stays for relatively long periods within one wing of the attractor before eventually switching to the other wing, thus, exhibiting persistent regime behavior. However, as we increase *r* the regime behavior survives but becomes less persistent. By increasing *r* both attractor wings are still present but the trajectory switches more frequently between them (Fig. 1b).

Analyzing the Lorenz 63 model by increasing *r* shows the impact of the regime behavior on the Hurst effect. For measuring the Hurst effect we have employed several methods: power spectral methods^17^ and Detrended Fluctuation Analysis (DFA, see Methods and Data)^22^,^23^. For computing DFA we use quadratic polynomial detrending (referred to as DFA2). Our results are insensitive to the order of the detrending.

We find that the standard case (*r* = 28) exhibits the Hurst effect with a Hurst exponent of 
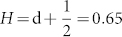
. The DFA2 scaling extends over almost 4 orders of magnitude with no sign of leveling off of the scaling on the longest timescales. This provides strong evidence for the Hurst effect in the Lorenz 63 model (Fig. 1).

**Figure 2:**
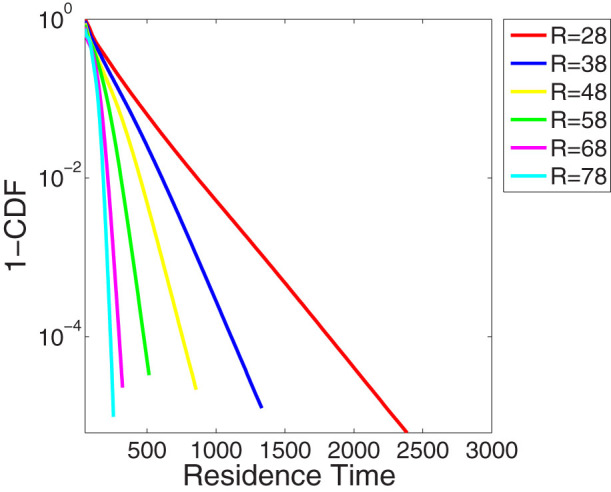
Cumulative distribution functions of the residence time for the Lorenz 63 model for various values of the r parameter. The residence time is measured in 0.1 time units.

DFA2 shows that experiments with *r* larger than 58 are however consistent with scaling expected from white noise (Fig. 1). Here we argue that the reduction in the magnitude of the Hurst exponent arises likely from the time the Lorenz 63 model resides continuously in one of the two regime states as shown in Fig. 2. This figure displays the complementary cumulative distribution of the residence time, i.e. of the length of the periods the system spends in one of the two regime states. This figure shows that for increasing *r* the residence time progressively decreases.

**Figure 3:**
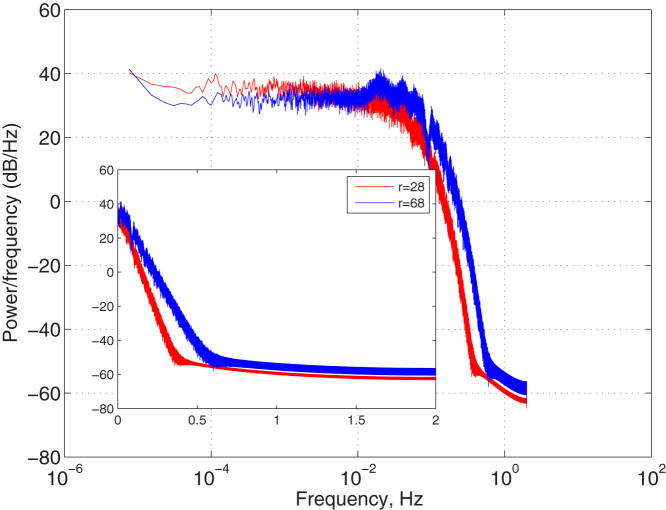
Double logarithmic power spectra of Lorenz 63 model with *r* = 28 (x variable) and *r* = 68. Inset shows plots on semilog axes. The time series is sampled in 0.1 time units.

To further check whether the Lorenz 63 model shows the Hurst effect we computed the power spectrum using the Welch periodogram method (see Methods and Data). The inset of Fig. 3 shows for high frequencies the exponential form for the periodogram reported by several previous authors^24^,^25^,^26^. However, in the main plot of Fig. 3 we can see that in the *r* = 68 case the power spectrum becomes flat for low frequencies and is thus consistent with white noise and inconsistent with a Hurst effect, whereas for *r* = 28 an upward trend towards the lowest frequencies is clearly seen, consistent with the Hurst effect reported by DFA. We get similar results when using the GPH estimator (see Methods and Data)^27^.

As shown in Fig. 2 the residence time decays exponentially. This indicates a memory-less switching process between the two regime states in the Lorenz 63 model consistent with the study by Aizawa^28^. Such a process has a mean regime residence time *τ_Res_* in one of the attractor wings (Tab. 1). This residence time is significantly longer than the Lyapunov time scale. It is frequently assumed that the Lyapunov time scale determines the limit of predictability, and that beyond these time scales systems are effectively characterized by white noise; i.e. are not predictable. The largest Lyapunov exponent provides an estimate for the rate of separation of two trajectories which are initially infinitesimally close to each other. We find that the time scale associated with the largest Lyapunov exponent of the Lorenz 63 model is significantly shorter than the time scales over which we find the Hurst effect occurring (i.e. *τ_L_* < *τ_Res_* (Tab. 1)). This challenges the idea that deterministic chaotic systems are necessarily unpredictable on time scales much longer than the Lyapunov time scale *τ_L_*. Our results are consistent with the idea that the predictability of multi-scale systems can be enhanced beyond the Lyapunov time scale^20^. Our results provide evidence that the Lorenz 63 model can be seen as a system with multiple or a continuum of time scales and has not just one intrinsic time scale. The multiple time scales are due to the chaotic regime switching which occurs randomly.

As with all time series analysis our results come with the caveat of finite size effects. While we cannot rule out that on much longer time scales the slope will eventually approach 0.5, i.e. iid white noise, we find strong evidence for the Hurst effect in the Lorenz 63 model because the scaling extends over 4 orders of magnitude, and is confirmed by spectral analysis. Furthermore, our time series lengths are comparable with the lengths of many observed natural time series. Thus, our results clearly apply to the interpretation of observed time series. The growth of range on these long time scales due to regime behavior causes significant impacts on predictability, extremes and trends^29^,^30^.

## The Hurst Effect in the Atmosphere

We have shown that the deterministic chaotic Lorenz 63 model exhibits the Hurst effect, and that the regime behavior is its cause. Similar regime behavior exists in the atmosphere and we assert that this is key for explaining the Hurst effect in the atmosphere. The basic idea of this stems from econometrics^15^. Diebold and Inoue^15^ showed both theoretically and by Monte Carlo simulations that Markov-switching models are able to produce the Hurst effect when the corresponding Markov transition matrix is metastable^31^. This corresponds to persistent regime behavior where the system stays close to one region in phase space before it eventually switches to another region to stay in that region for a long period. Such a behavior is ubiquitous in the atmosphere^32^,^33^,^34^,^35^.

To identify the presence of the Hurst effect in the atmosphere, we analyze the North Atlantic jet stream in both reanalysis and climate model simulations (see Methods and Data), whose variations greatly impact European climate. We use the Jet Latitude Index (JLI) as a measure of the North Atlantic jet stream^36^. In Franzke^30^ it has been argued that the regime behavior of the JLI is responsible for the Hurst effect of surface wind speeds. Here we show evidence that even the JLI exhibits the Hurst effect (Fig. 1c). The JLI shows scaling over up to two order of magnitude. The JLI has a Hurst exponent of *H* = 0.59 in the reanalysis data. Franzke et al.^35^ showed that the regime behavior is consistent with an eddy-mean flow feedback due to wave breaking. Thus, this nonlinear deterministic behavior is intimately associated with the Hurst effect. This provides a new physical mechanism for explaining the Hurst effect in the atmosphere, which has hitherto been lacking.

A fact which hasn't been widely appreciated is that persistent jet states are self-maintaining^37^. This self-maintenance arises from eddy-mean flow feedbacks and Rossby wave breaking, which are fundamental properties of geophysical flows. Hence, regime behavior and the Hurst effect are intrinsic properties of geophysical flows and the atmosphere. Hence, it is important that climate models capture this phenomenon in order for us to have confidence in their ability to predict future climate.

**Figure 4:**
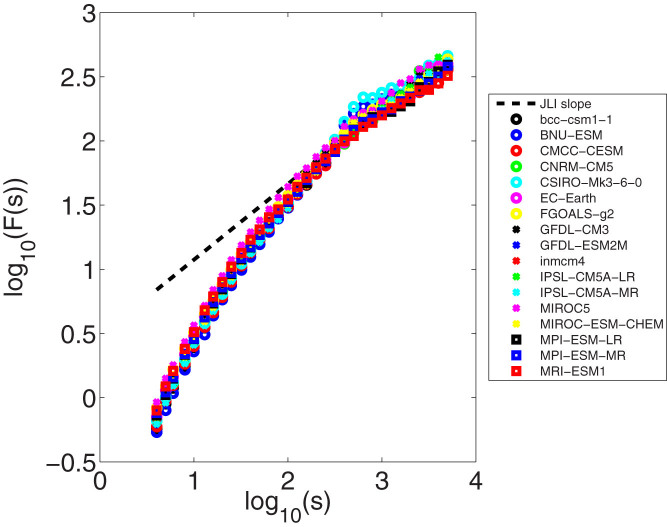
DFA spectra of CMIP5 JLIs. The JLI time series are sampled daily.

We now evaluate whether the current generation of climate models (see Methods and Data) reproduce the Hurst effect characteristics of the observed JLI in reanalysis data. For this we use the historical simulations from the CMIP5 archive^38^. As Fig. 4 shows, most CMIP5 models exhibit the Hurst effect with roughly the right magnitude (Tab. 2). Most CMIP5 models show scaling over up to two orders of magnitude. While the CMIP5 models don't capture the shape of the observed JLI PDF^39^ they still seem to capture the essential atmospheric dynamics of the jet stream. Recent studies show that very high resolutions are needed to accurately reproduce the geographical structure of the atmospheric flow regimes^34^. However, the CMIP5 models already capture the important geophysical dynamics and, thus, the scaling behavior.

## Discussion

Our results clearly show that the Hurst effect is not necessarily synonymous with stationary LRD. We showed that a deterministic chaotic system, the Lorenz 63 model, can exhibit the Hurst effect due to its non-stationary regime behavior. This regime behavior can also be seen as a kind of intermittency which can also create power laws^40^. Because the residence time decays exponentially and not like a power law this questions whether the Lorenz 63 model has infinite memory. Typically, systems exhibiting LRD have power-law distributed waiting times. This interpretation of our results is consistent with previous studies^15^ who have shown that Markov switching models (which have regime behavior) or more general non-stationary models^13^ produce growth of range without infinite memory, at least over large ranges of scale.

Many climate scientists are deeply sceptical about LRD on theoretical grounds^41^,^42^, and have transferred this scepticism to the Hurst effect in the belief that it necessarily corresponds to an infinite memory process. But our results show that in a climate-relevant dynamical system, the Hurst effect can arise from well understood nonlinear dynamics.

The wider implications of our results are that regime behavior impacts on trend analysis because the switching can cause apparent trends by e.g. staying in the first half of the time series in one regime state and then switching to another regime state for the second half (see Fig. 1b of Ref. 29 for an illustration of this effect). Furthermore, we showed that the Hurst effect can be an intrinsic property of nonlinear dynamical systems. Our ideas put forward here are consistent with the work by Ed Lorenz on climate as an almost intransitive system^43^. His work suggests that even deterministic systems, when they are nonlinear, can exhibit variability on very long time scales. Our results on the Hurst effect in climate are consistent with his ideas.

Moreover, our results have implications for future climate projections. We show that on long (but finite) timescales, the Lorenz 63 system exhibits the Hurst effect for lower Raleigh parameter values; physically this denotes a reduced temperature difference between plates in a Rayleigh-Benard experiment. We see in Lorenz 63 that if we make the system less chaotic, the trajectory stays in a given wing for longer with only inter-mittent sojourns to the other wing. From these results we can make an analogy with the weather conditions affecting Europe and the US in the last few years. We expect a reduced meridional temperature gradient due to declining sea ice extent. We anticipate that this ought to make the (lower) atmosphere more stable (less chaotic) to baroclinic instability. In a system which has regimes this can be seen by greater residence times for a given regime. Many of the recent extreme weather conditions can be linked to unusual and persistent deviations of the jet stream^44^, though their significance and relevance has been questioned^45^. Our results suggest that the Hurst effect characteristics of the atmosphere might be more important in explaining current extreme weather conditions than previously thought.

## Methods

In this study we use data from climate models which has also been used for the latest, Fifth Assessment Report (AR5) of the Intergovernmental Panel on Climate Change (IPCC). The data are from the Coupled Model Intercomparison Project - Phase 5 (CMIP5) which includes a variety of different standardized experiments performed with state-of-the-art general circulation models^38^. According to the availability on the online archive (http://pcmdi3.llnl.gov/esgcet/home.htm), historical runs for 25 different climate models spanning over the 1951–2005 period (55 full years) were analyzed. The ensemble member r1i1p1 has been selected for all models included except for the Community Climate System Model (CCSM4; simulation r6i1p1) and the European Centre Earth System Model (EC-EARTH; simulation r8i1p1). The list of the models chosen is shown in Tab. 2. See Ref. 38 for more details about the climate model simulations. We use data from the following modelling groups: Beijing Climate Center (China, BCC-CSM1-1), Beijing Normal University (China, BNU-ESM), Centro Euro-Mediterraneo sui Cambiamenti Climatici (Italy, CMCC-CESM), Meteo-France (France, CNRM-CM5), Commonwealth Scientific and Industrial Research Organisation (Auistralia, CSIRO-Mk3-6-0), European Network for Earth System Modelling (EC-EARTH), State Key Laboratory Numerical Modeling for atmospheric Sciences and geophysical fluid Dynamics (China, FGOALS-g2), Geophysical Fluid Dynamics Laboratory (USA, GFDL-CM3 and GFDL-ESM2M), Russian Academy of Sciences Institute of Numerical mathematics (Russia, inmcm4), Institut Pierre Simon Laplace (France, IPSL-CM5A-LR and IPSL-CM5A-MR), International Centre for Earth Simulation (Japan, MIROC5 and MIROCESM-CHEM), Max-Planck Institute for Meteorology (Germany, MPI-ESM-LR and MPI-ESM-MR) and Meteorological Research Institute (Japan, MRI-ESM1). More information about the CMIP5 climate models and the data can be found at: http://esgf-data.dkrz.de/esgf-web-fe/.

The Jet Latitude Index (JLI)^36^ is introduced in order to describe the daily variability of the latitudinal position of the eddy-driven Atlantic jet stream. The JLI is defined as the latitude of the zonally averaged maximum of the zonal wind speed between 60°W and 0°W longitude at 850 hPa. A 5-day running-mean is applied in order to exclude synoptic variability. Model data are interpolated on the 2.5°× 2.5° grid using a second order conservative remapping algorithm. Only values between 15°N and 75°N are retained to avoid orographic effects. The CMIP5 JLI index is slightly differently defined from Woollings et al.^36^ but both versions are highly correlated. Furthermore, also a JLI derived from the 20th Century National Center for Environment Prediction (20C NCEP) reanalysis data show very similar results to the European Centre for Medium-Range Weather Forecasts ERA40 JLI results. This provides further evidence that our results are robust.

In order to examine the Hurst effect we use DFA^8^,^22^,^41^,^46^. In DFA, first a profile 
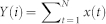
 is computed. This profile is then split up into *N_s_* non-overlapping segments of equal length s. After that the local trend is subtracted for each segment v by a polynomial least-squares fit of the data. Linear (DFA1), quadratic (DFA2), cubic (DFA3) or higher-order polynomials can be used for detrending. In the nth-order DFA, trends of order n in the profile, and of order n-1 in the original record, are eliminated. Next, the variance for each of the *N_s_* segments is calculated by averaging over all data points i in the v-th segment:
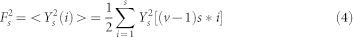


Finally, the average over all segments is computed and the square root is applied to obtain the following fluctuation function:
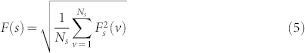


For different detrending orders, n, we obtain different fluctuation functions F (s), which are denoted by *F*^(*n*)^ ~ (*s*). The fluctuation function scales according to *F*^(*n*)^(*s*) ~ *s^ζ^*, with *ζ* corresponding to the Hurst exponent H and 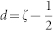.

We also use the Welch method to estimate the spectral density of the time series. This method reduces the variance of the periodogram by splitting the time series into overlapping segments. For each segment the periodogram is computed and these estimates are averaged.

Furthermore, we use also a power spectral estimator to infer the Hurst exponent. In particular, we are using the GPH estimator^27^,^46^. This estimatior finds the Hurst exponent by estimating the spectral slope using the periodogram.

## Author Contributions

C.F. and S.O. designed the study, C.F., S.O., P.D. and N.W. carried out the research. C.F., S.O., P.D. and N.W. wrote the manuscript.

## Figures and Tables

**Table 1:** Hurst exponent (*H*(DFA), Lyapunov time (*τ_L_*) and mean regime residence time (*τ_Res_*) of the x-component of Lorenz 63 model for various values of the Rayleigh parameter r

R	*H*(DFA)	*τ_L_*	*τ_Res_*
28	0.65	1.09	19.0
38	0.62	0.91	15.4
48	0.56	0.81	12.5
58	0.50	0.74	10.9
68	0.49	0.68	9.1
78	0.48	0.65	8.7
88	0.49	0.65	6.6

**Table 2:** Hurst exponent *H* values of JLI from CMIP5 historical forcing runs covering the period 1951 through 2005. Hurst exponents have been computed by the GPH and DFA2 estimators. The GPH estimator also provides the 5% confidence levels

Model	*H*(GPH)	*H*(DFA2)
BCC-CSM1-1	0.62 ± 0.06	0.57
BNU-ESM	0.68 ± 0.06	0.54
CMCC-CESM	0.64 ± 0.06	0.55
CNRM-CM5	0.62 ± 0.06	0.62
CSIRO-Mk3-6-0	0.66 ± 0.06	0.59
EC-Earth	0.61 ± 0.06	0.58
FGOALS-g2	0.61 ± 0.06	0.55
GFDL-CM3	0.66 ± 0.06	0.57
GFDL-ESM2M	0.63 ± 0.06	0.55
inmcm4	0.63 ± 0.06	0.55
IPSL-CM5A-LR	0.63 ± 0.06	0.61
IPSL-CM5A-MR	0.64 ± 0.06	0.59
MIROC5	0.63 ± 0.06	0.55
MIROC-ESM-CHEM	0.61 ± 0.06	0.57
MPI-ESM-LR	0.64 ± 0.06	0.56
MPI-ESM-MR	0.62 ± 0.06	0.55
MRI-ESM1	0.59 ± 0.06	0.51
